# Preoperative assessment of tertiary lymphoid structures in stage I lung adenocarcinoma using CT radiomics: a multicenter retrospective cohort study

**DOI:** 10.1186/s40644-024-00813-5

**Published:** 2024-12-18

**Authors:** Xiaojiang Zhao, Yuhang Wang, Mengli Xue, Yun Ding, Han Zhang, Kai Wang, Jie Ren, Xin Li, Meilin Xu, Jun Lv, Zixiao Wang, Daqiang Sun

**Affiliations:** 1 Chest hospital, Tianjin University, Tianjin, China; 2 Department of Thoracic Surgery, Tianjin Chest Hospital, No. 261, Taierzhuang South Road, Jinnan District, Tianjin, 300222 China; 3 Clinical School of Thoracic, Tianjin Medical University, Tianjin, China; 4 Department of Pathology, Tianjin Chest Hospital, Tianjin, China; 5 Department of Thoracic Surgery, Tianjin Jinnan Hospital, Tianjin, China; 6 Department of Imaging, Tianjin Chest Hospital, Tianjin, China; 7 Department of Thoracic Surgery, Qinhuangdao First Hospital, Hebei Province, China

**Keywords:** Lung adenocarcinoma, Tertiary lymphoid structures, Radiomics, Deep learning

## Abstract

**Objective:**

To develop a multimodal predictive model, Radiomics Integrated TLSs System (RAITS), based on preoperative CT radiomic features for the identification of TLSs in stage I lung adenocarcinoma patients and to evaluate its potential in prognosis stratification and guiding personalized treatment.

**Methods:**

The most recent preoperative chest CT thin-slice scans and postoperative hematoxylin and eosin-stained pathology sections of patients diagnosed with stage I LUAD were retrospectively collected. Tumor segmentation was achieved using an automatic virtual adversarial training segmentation algorithm based on a three-dimensional U-shape convolutional neural network (3D U-Net). Radiomic features were extracted from the tumor and peritumoral areas, with extensions of 2 mm, 4 mm, 6 mm, and 8 mm, respectively, and deep learning image features were extracted through a convolutional neural network. Subsequently, the RAITS was constructed. The performance of RAITS was then evaluated in both the train and validation cohorts.

**Results:**

RAITS demonstrated superior AUC, sensitivity, and specificity in both the training and external validation cohorts, outperforming traditional unimodal models. In the validation cohort, RAITS achieved an AUC of 0.78 (95% CI, 0.69–0.88) and showed higher net benefits across most threshold ranges. RAITS exhibited strong discriminative ability in risk stratification, with *p* < 0.01 in the training cohort and *p* = 0.02 in the validation cohort, consistent with the actual predictive performance of TLSs, where TLS-positive patients had significantly higher recurrence-free survival (RFS) compared to TLS-negative patients (*p* = 0.04 in the training cohort, *p* = 0.02 in the validation cohort).

**Conclusion:**

As a multimodal predictive model based on preoperative CT radiomic features, RAITS demonstrated excellent performance in identifying TLSs in stage I LUAD and holds potential value in clinical decision-making.

**Supplementary Information:**

The online version contains supplementary material available at 10.1186/s40644-024-00813-5.

## Key points

• Accurately predicting tertiary lymphoid structures (TLSs) in stage I lung adenocarcinoma is crucial for identifying high-risk patients and guiding personalized treatment strategies for early-stage lung adenocarcinoma.

• The multimodal model, the radiomics integrated TLSs system (RAITS), had an AUC of 0.78 (95% CI, 0.69–0.88) in the test set, and its predictive efficacy was better than that of the traditional unimodal model.

• The RAITS has the potential to be a well-established tool for noninvasive assessment of TLSs, guiding therapeutic strategies and facilitating adjuvant therapy programs.

**Supplementary Information:**

The online version contains supplementary material available at 10.1186/s40644-024-00813-5.

## Introduction

Lung cancer, as one of the most prevalent malignancies worldwide, constitutes a leading cause of cancer-related mortality [1]. Lung adenocarcinoma (LUAD) is a primary histological subtype of lung cancer. Studies have found that even after completing surgical resection, stage I LUAD can have a recurrence rate as high as 20–50%. Late-stage tumors are often diagnosed as unresectable, with a median survival period of less than 12 months, and carry high risks of recurrence and metastasis, resulting in a poor prognosis [2–4]. Therefore, promptly identifying high-risk factors and adjusting treatment plans are crucial for reducing recurrence rates in early-stage LUAD, prolonging survival in late-stage patients, and improving overall prognosis.

In recent years, tumor-associated tertiary lymphoid structures (TLSs) have gained widespread attention due to their potential value in prognosis assessment and guiding immunotherapy. Recent studies have indicated that TLSs play a significant role in predicting cancer prognosis and adjusting adjuvant treatment strategies [5, 6]. Sun et al.‘s research [7]showed that in resectable non-small cell lung cancer (NSCLC) patients receiving neoadjuvant immunochemotherapy, the maturity and abundance of TLSs were significantly increased and notably associated with major pathological response (MPR) and pathological complete response (pCR). Furthermore, studies have shown that the presence of TLSs is associated with a better prognosis in cancer patients, and those with high TLS maturity and abundance exhibit improved disease-free survival (DFS) [8]. Collectively, these studies suggest that by assessing the presence and characteristics of TLSs, clinicians can effectively predict patient responses to immunotherapy, thereby adjusting treatment plans and selecting more appropriate immunotherapy strategies.

In recent years, with the rapid development of machine learning and artificial intelligence (AI) technologies in the medical field, researchers have begun utilizing these technologies to extract and analyze quantitative data from medical images that are imperceptible to the naked eye, thereby supporting disease diagnosis and prognosis assessment. Research indicates that utilizing radiomic features of tumors and surrounding tissues can effectively predict the type, prognosis, and pathological subtypes of LUAD, facilitating better clinical decision-making [9–11]. However, as of the writing of this article, no studies have been published on predicting TLSs in early-stage LUAD based on radiomic features of tumors and surrounding tissues. Consequently, we intend to develop methods to predict the presence of TLSs using radiomic features derived from chest computed tomography (CT) scans, enabling accurate preoperative identification of TLSs to guide adjustments in treatment strategies.

In summary, we have developed and validated the radiomics integrated TLSs system (RAITS) in this multicenter retrospective cohort study, leveraging preoperative chest CT deep learning and radiomic features. This system assists clinicians in the early identification of TLSs in LUAD, thereby facilitating timely adjustments in patient treatment strategies and potentially improving patient outcomes.

## Methods and materials

### Study design and patients

**Fig. 1:**
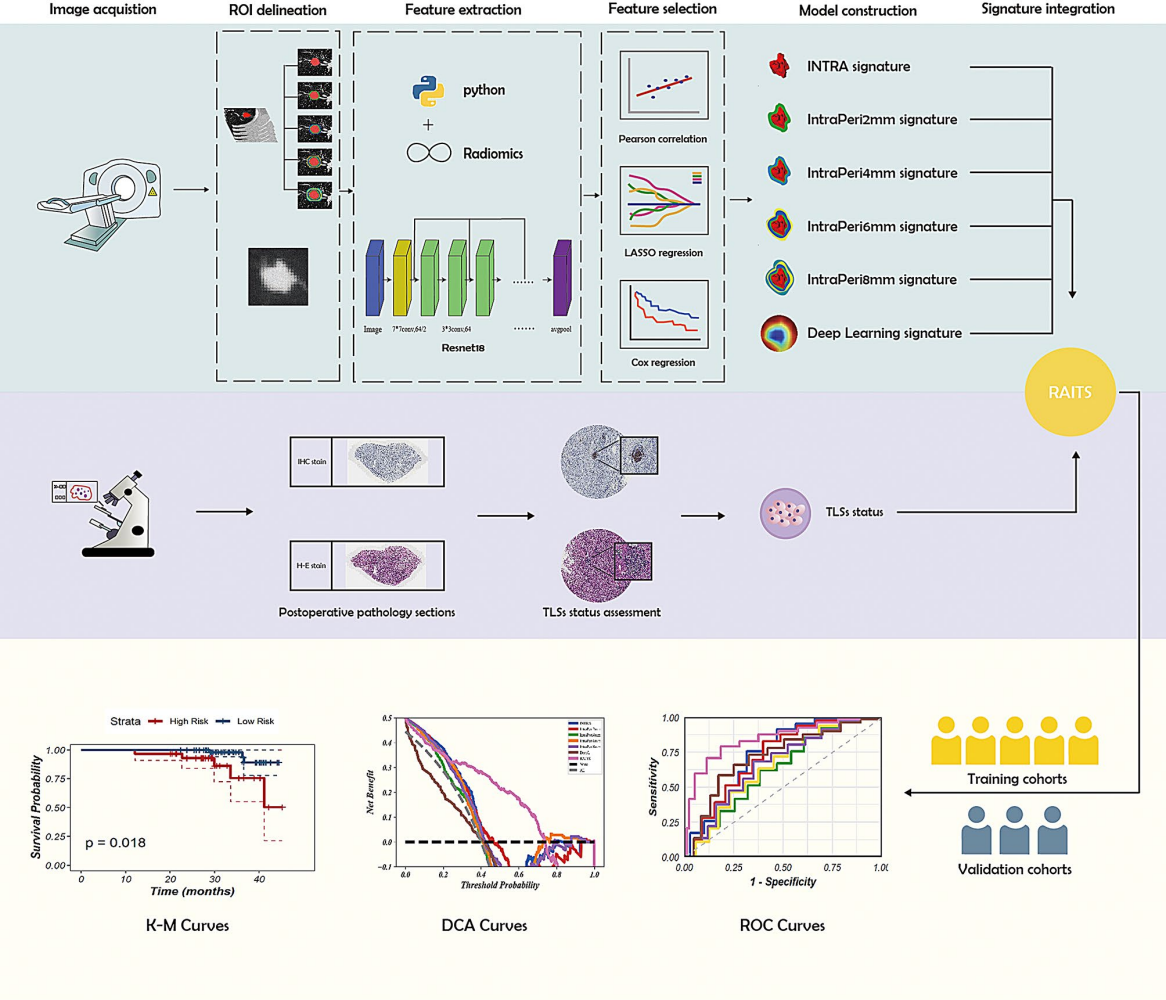
The workflow Diagram

**Fig. 2:**
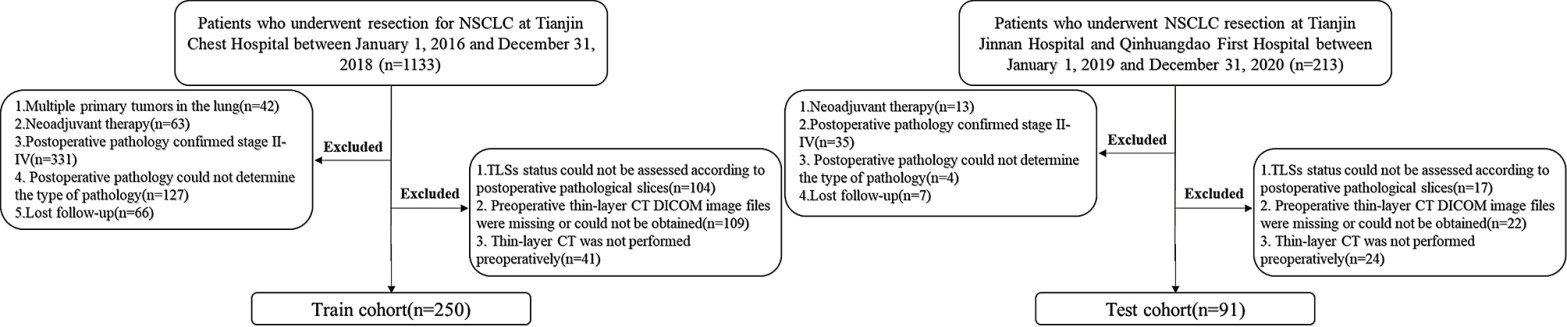
Patient inclusion and exclusion criteria

This study was approved by the ethics committee, and due to its retrospective nature, informed consent was not required. The study primarily consists of two parts: the development of the model and its external validation, both using retrospective data. The study was divided into four main steps: image acquisition and processing, pathological assessment of TLSs, feature extraction and selection, and model construction and evaluation (Fig. 1).Information on patients treated at Tianjin Chest Hospital from January 1, 2016, to December 31, 2018, was reviewed, and patients who met the following inclusion criteria were included: (1) those who underwent complete surgical resection of lung lesions at Tianjin Chest Hospital; (2) postoperative pathology confirmed invasive stage I LUAD. The exclusion criteria were as follows: (a) multiple primary lung cancers; (b) preoperative neoadjuvant therapy; (c) loss to follow-up after surgery; (d) inability to assess the status of TLSs from postoperative pathological slides; (e) missing, inaccessible, or unavailable preoperative thin-slice CT files. After screening, 250 eligible patients were ultimately included in the study as the training cohort. Using the same inclusion and exclusion criteria, the information and imaging data of patients treated at Tianjin Jinnan Hospital or Qinhuangdao First Hospital from January 1, 2019, to December 31, 2020, were screened. A total of 91 eligible patients were included in the study as the external validation cohort. Figure 2 illustrates the flowchart of the inclusion and exclusion criteria used in this study.

### CT scanning protocol and tumor segmentation

**Fig. 3:**
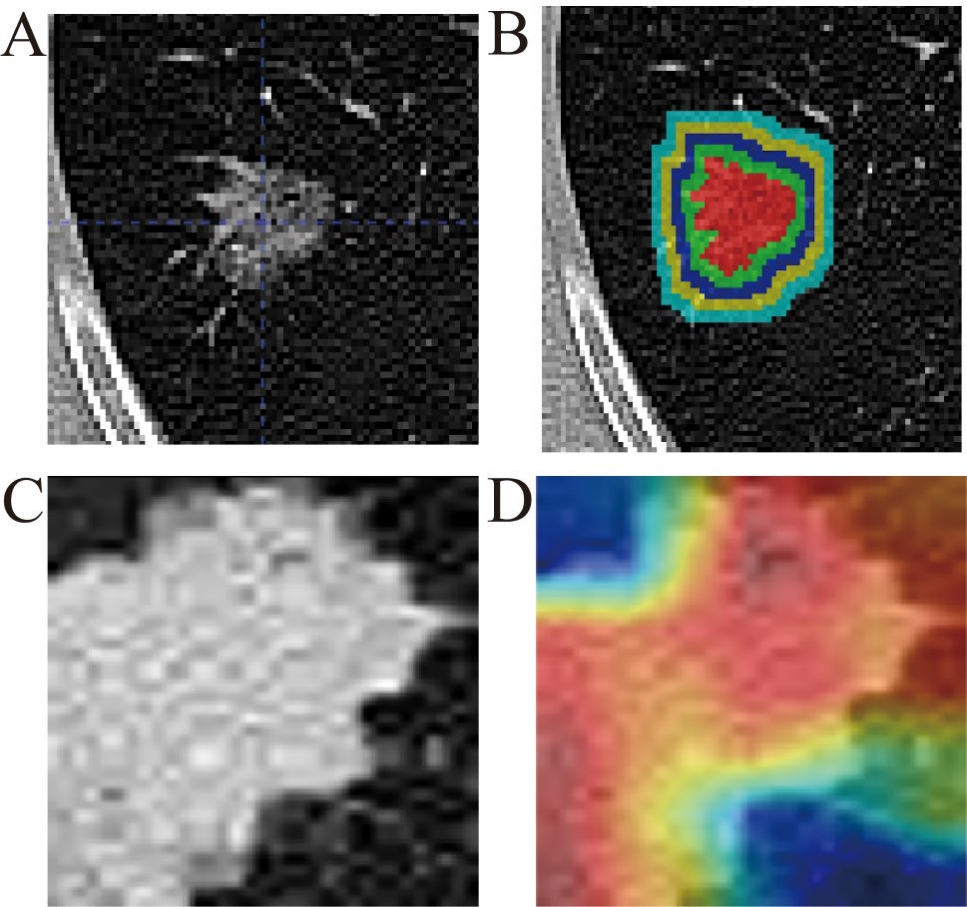
Region of interest(ROI) segmentation **(A)** Localization of the ROI. **(B)** Expansion of the tumor ROI on CT images by 2 mm, 4 mm, 6 mm, and 8 mm, creating the IntraPeri2mm, IntraPeri4mm, IntraPeri6mm, and IntraPeri8mm regions based on the tumor. **(C**-**D)** Extraction of the maximum cross-section of the ROI and deep learning feature extraction using a convolutional neural network (CNN). Visualization of the Gradient-weighted Class Activation Mapping (Grad-CAM) for a representative sample (‘114’)

The most recent preoperative chest CT images were downloaded in Digital Imaging and Communications in Medicine (DICOM) format from the Picture Archiving and Communication System (PACS) of Tianjin Chest Hospital, Tianjin Jinnan Hospital, and Qinhuangdao First Hospital. Due to the use of different CT scanners (including PNMS, Siemens, and Philips), variations in slice thickness, voxel size, window width, and window level existed among patients, necessitating preprocessing of the CT images. All CT images were resampled to standardize the voxel size to 0.7 mm × 0.7 mm × 1.5 mm, and the window width and window level were standardized to 1350 HU and − 350 HU, respectively. Tumor region segmentation was performed using the automatic Virtual Adversarial Training (VAT) segmentation algorithm based on a three-dimensional U-Net (3D U-Net) topology. The U-Net structure consists of five levels of encoding and decoding sub-networks. In each stage, the encoding part includes two convolutional layers and a max-pooling layer to compress spatial information. The decoding part contains two convolutional layers and a transpose convolution layer, which are responsible for reconstructing the segmented features (see Supplementary Fig. 1). The region of interest (ROI) segmentation was expanded to four peritumoral areas. After reviewing existing studies, peritumoral expansion regions of 2 mm, 4 mm, 6 mm, and 8 mm around the tumor were selected (Fig. 3B).

### Histological assessment

According to the World Health Organization’s (WHO) first formal definition of TLSs in 2018 and the study by MAN et al. [12], TLSs are defined as lymphoid aggregates of B lymphocytes, typically mixed with varying proportions of plasma cells and T lymphocytes. In this study, tumors were defined as TLS-positive if at least one TLS was observed within the tumor on Hematoxylin and Eosin (H&E) stained sections; otherwise, they were defined as TLS-negative. Additionally, under immunohistochemistry (IHC), TLSs can be further identified by labeling specific immune cell markers: CD20 for B cells, CD3 for T cells, and CD138 for plasma cells. Based on previous studies, TLSs were classified into low-maturity and high-maturity categories depending on the presence of CD21 + follicular dendritic cells. The total number of TLSs and their maturity were recorded under an optical microscope. Tumors were further divided into intratumoral (within 1 mm of the tumor boundary) and peritumoral (greater than 1 mm from the tumor boundary) regions, as a 1 mm distance is a relatively easy metric to determine. The number of TLSs in both regions was recorded [13, 14]. The exclusion criteria included: (1) lymphocyte aggregates containing fewer than 50 cells; (2) aggregates not exhibiting typical curved and smooth contours; (3) aggregates located in fibrous tissue more than 1 mm away from tumor cells; (4) failure to identify immune cell markers characteristic of TLSs in immunohistochemical staining.

**Fig. 4:**
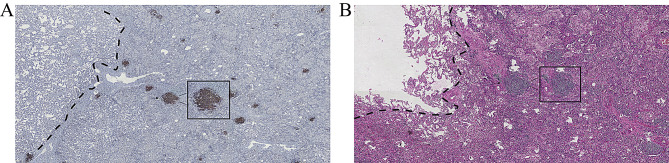
Microscopic images of TLSs. **(A)** Assessment of TLSs status in immunohistochemistry (IHC) sections. **(B)** Assessment of TLSs status in Hematoxylin and Eosin (H&E) stained sections

Postoperative H&E-stained sections and some IHC sections of surgical specimens from all enrolled patients were reviewed by pathologists X.M.L. (with 28 years of experience in pathology) and X.M.L. (with 3 years of experience in pathology) to determine the presence, number, location, and maturity of TLSs (Fig. 4). The pathologists were blinded to the patients’ prognoses. In the training cohort, 108 patients (43.2%) were identified as TLS-positive, and in the validation cohort, 30 patients (32.9%) were identified as TLS-positive.

### Follow-up

Prognostic information for all enrolled patients was obtained through the electronic medical record system and telephone follow-up. The endpoints of this study were recurrence-free survival (RFS) and tumor recurrence. Tumor recurrence was confirmed through imaging or lymph node pathology using fine-needle aspiration biopsy and bronchoscopy to identify local recurrence or distant metastasis. The last follow-up date for patients in the training cohort was September 1, 2021, and for those in the validation cohort, it was May 1, 2024.

### Deep learning feature extraction

In the deep learning image processing, the slice showing the largest ROI was selected as the representative image for each patient (Fig. 3C). In this study, the well-known neural network ResNet18 was used to extract deep learning features. Transfer learning was implemented to ensure the model’s effectiveness across diverse patient populations with significant differences. The learning rate was adjusted to achieve better generalization across different datasets, and a cosine decay learning rate strategy was adopted, defined as follows:

$$\:{\eta\:}_{t}={\eta\:}_{min}^{i}+\frac{1}{2}\left({\eta\:}_{max}^{i}-{\eta\:}_{min}^{i}\right)\left(1+cos\left(\frac{{T}_{cur}}{{T}_{i}}\pi\:\right)\right)$$

$\:{\eta\:}_{min}^{i}=0$ represents the minimum learning rate, $\:{\eta\:}_{max}^{i}=0.01$ represents the maximum learning rate, and $\:{T}_{i}=30$ indicates the number of epochs in the iterative training process. In our signature, the output probabilities computed by the CNN were defined as deep learning features.

### Radiomics model construction

Feature extraction used Pyradiomics according to IBSI guidelines. Features with significant group differences were retained through t-tests and correlation tests. LASSO regression reduced irrelevant features, with lambda optimized via cross-validation. Models were evaluated using receiver operating characteristic (ROC) and decision curve analysis (DCA) curves.

### Statistical analysis

Continuous variables in the baseline data that follow a normal distribution are expressed as mean ± standard deviation (SD). Continuous variables that do not follow a normal distribution are expressed as median (interquartile range). The DeLong test was used to assess variability between multiple models. High-risk and low-risk groups were identified based on the optimal cutoff value of the final model derived from the ROC curve. Survival curves were plotted using the Kaplan-Meier method. A p-value of less than 0.05 was considered statistically significant. All statistical analyses were performed using R software (version 4.2.2).

## Results

### Clinical characteristics of patients and TLSs evaluation

The baseline characteristics of the 250 patients in the training cohort and the 91 patients in the validation cohort are presented in Table 1. The overall distribution of TLS-positive and TLS-negative cases in the training and validation cohorts was compared. The results indicated no significant differences between the two cohorts. In the training cohort, the total number of TLSs in positive patients was 4.04 ± 2.74; the intratumoral number was 1.95 ± 1.68; the peritumoral number was 2.07 ± 2.16; and the mature number was 1.38 ± 1.41.In the validation cohort, the total number of TLSs in positive patients was 6.1 ± 2.54; the intratumoral number was 2.77 ± 2.27; the peritumoral number was 3.33 ± 1.77; and the mature number was 0.93 ± 1.17.Detailed clinical data are presented in Supplementary Table 1.

**Table 1:** Baseline clinical characteristics of patients

Characteristics	Train cohort(250)			Validation cohort(91)		
	TLS-positive	TLS-negative	*P* value	TLS-positive	TLS-negative	*P* value
Age (Mean ± SD)	60.72 ± 10.08	62.5 ± 8.11	0.135	59.57 ± 8.54	59.79 ± 9.64	0.912
Gender n, (%)			0.386			0.547
Male	54 (50)	80 (56.3)		12 (40%)	19 (31.1%)	
Female	54 (50)	62 (43.7)		18 (60%)	42 (68.9%)	
Smoke n, (%)			0.095			1
Yes	37 (34.3)	66 (46.5)		11 (36.7%)	22 (36.1%)	
No	71(65.7)	76 (53.5)		19 (63.3%)	39 (63.9%)	
TNM stage n, (%)			0.300			0.885
IA	68 (63.0)	79 (55.6)		15 (50%)	33 (54.1%)	
IB	40 (37.0)	63 (44.4)		15 (50%)	28 (45.9%)	
TLSs Number (Mean ± SD)	4.04 ± 2.74	-	-	6.1 ± 2.54	-	-
TLSs Intra (Mean ± SD)	1.95 ± 1.68	-	-	2.77 ± 2.27	-	-
TLSs Peri (Mean ± SD)	2.07 ± 2.16	-	-	3.33 ± 1.77	-	-
TLSs Mature (Mean ± SD)	1.38 ± 1.41	-	-	0.93 ± 1.17	-	-

TLSs, tertiary lymphoid structures; SD, standard deviation; Intra, intra-tumor area; Peri, peri-tumor area

### Feature extraction and selection

For the tumor’s ROI on CT images (Fig. 3B), PyRadiomics was used to extract 1,834 features from the INTRA, IntraPeri2mm, IntraPeri4mm, IntraPeri6mm, and IntraPeri8mm regions. In the deep learning signature used in this study, the penultimate layer was utilized for feature extraction. To construct the RAITS features, the 300-dimensional deep learning features were combined with the 9,168-dimensional radiomics features. After feature selection, 8 highly correlated features were ultimately retained. (Supplementary Figs. 2–4). Details of the extracted features are provided in Supplementary Tables 2–2.

### RAITS evaluation

**Fig. 5:**
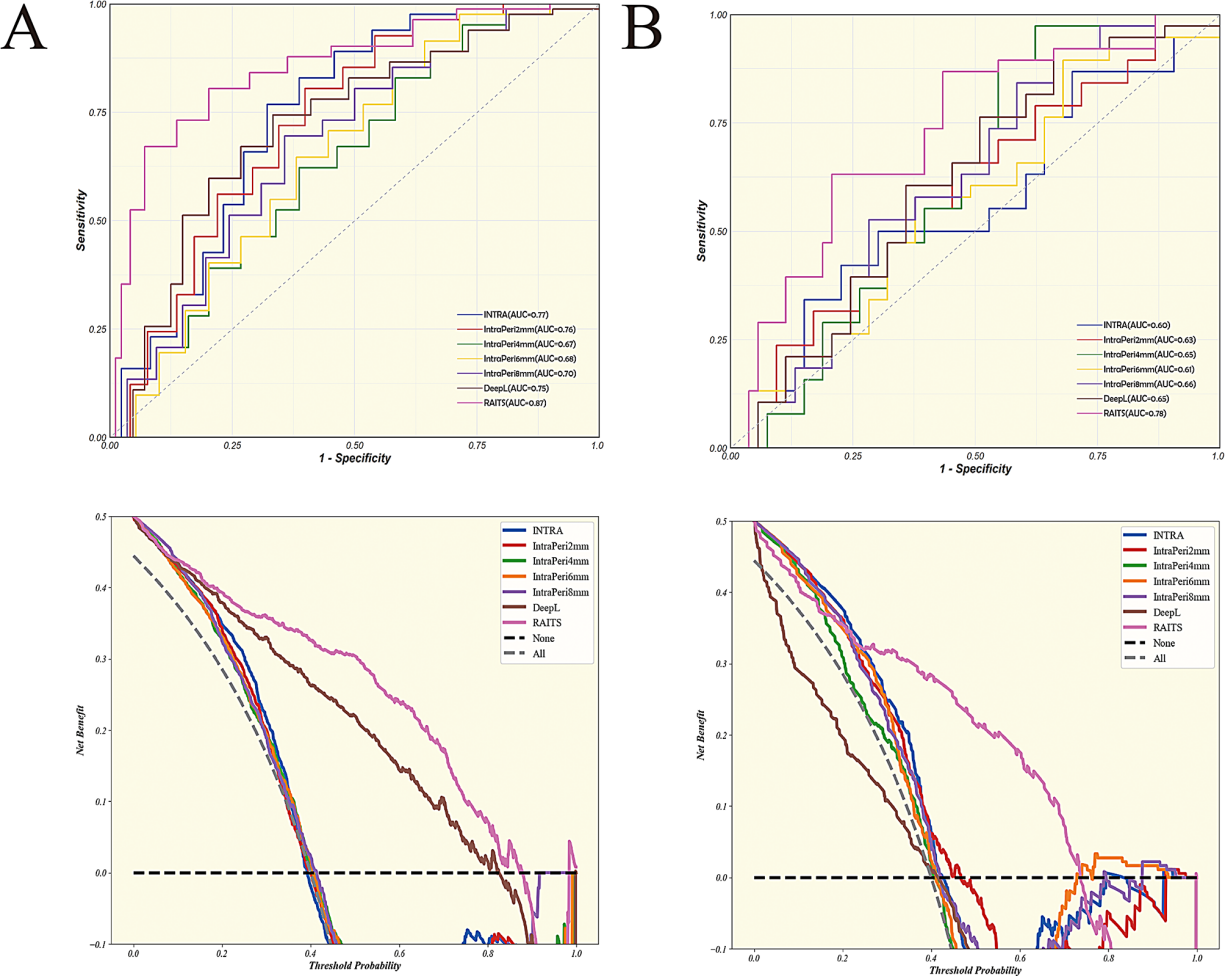
Performance of RAITS and unimodal models across all cohorts. **(A)** Receiver operating characteristic (ROC) and decision curve analysis (DCA) curves for RAITS and unimodal models in the training cohort. **(B)** ROC and DCA curves for RAITS and unimodal models in the validation cohort

The selected 8 variables were modeled using a LR model to construct RAITS. The performance of RAITS was evaluated in both the training and validation cohorts (Fig. 5; Table 2). RAITS achieved an AUC of 0.87 (95% confidence interval [CI], 0.82–0.92) in the training set and an AUC of 0.78 (95% CI, 0.69–0.88) in the validation set. The sensitivity and specificity of RAITS were 0.71 and 0.92 in the training set, and 0.66 and 0.83 in the validation set, respectively. The Youden index of RAITS was 0.63 in the training set and 0.49 in the validation set. Among all models, RAITS performed best in both the training and validation sets. A DeLong test was performed on the models in the validation set (Supplementary Table 10), demonstrating that RAITS had a significant advantage in predictive performance compared to IntraPeri6mm (0.78 vs. 0.61, *P* = 0.03). In the *P*-value matrix (Supplementary Fig. 5), the statistical significance of the AUC differences between various models was assessed, indicating that the AUC differences between RAITS and other models were statistically significant. The DCA graph illustrated the net benefit of different models at various thresholds. The results showed that RAITS demonstrated higher net benefits across most threshold ranges, both in the training and validation sets, indicating its substantial potential for application in clinical decision-making.

**Table 2:** Performance evaluation of the models in the training cohort and validation cohort

	AUC (95%CI)	Sensitivity	Specificity	PPV	NPV	Accuracy	Youden Index
**Training cohort**							
INTRA	0.77(0.71–0.83)	0.82	0.67	0.55	0.88	0.72	0.49
IntraPeri2mm	0.76(0.70–0.82)	0.84	0.60	0.51	0.89	0.68	0.44
IntraPeri4mm	0.67(0.60–0.74)	0.89	0.38	0.41	0.88	0.55	0.27
IntraPeri6mm	0.68(0.62–0.75)	0.83	0.48	0.44	0.85	0.59	0.31
IntraPeri8mm	0.70(0.64–0.77)	0.67	0.68	0.51	0.81	0.68	0.36
DeepL	0.75(0.69–0.81)	0.60	0.83	0.63	0.81	0.75	0.42
RAITS	0.87(0.82–0.92)	0.71	0.92	0.82	0.87	0.85	0.63
**validation cohort**							
INTRA	0.60(0.48–0.72)	0.42	0.83	0.64	0.67	0.66	0.25
IntraPeri2mm	0.63(0.52–0.75)	0.55	0.68	0.55	0.68	0.63	0.23
IntraPeri4mm	0.65(0.54–0.76)	0.92	0.43	0.54	0.88	0.64	0.36
IntraPeri6mm	0.61(0.49–0.72)	0.55	0.68	0.55	0.68	0.63	0.23
IntraPeri8mm	0.66(0.54–0.77)	0.89	0.40	0.52	0.84	0.60	0.29
DeepL	0.65(0.54–0.76)	0.76	0.51	0.53	0.75	0.62	0.27
RAITS	0.78(0.69–0.88)	0.66	0.83	0.74	0.77	0.76	0.49

AUC, area under the receiver operating characteristics curve; CI, confidence interval; PPV, positive predictive values; NPV, negative predictive values; INTRA, intra-tumoral signature; IntraPeri2mm, peritumoral radiomic signature extracted from the 2 mm peritumoral area; IntraPeri4mm, peritumoral radiomic signature extracted from the 4 mm peritumoral area; IntraPeri6mm, peritumoral radiomic signature extracted from the 6 mm peritumoral area; IntraPeri8mm, peritumoral radiomic signature extracted from the 8 mm peritumoral area; DeepL, radiomics signature constructed by deep learning features; RAITS, Radiomics Integrated TLSs System

**Fig. 6:**
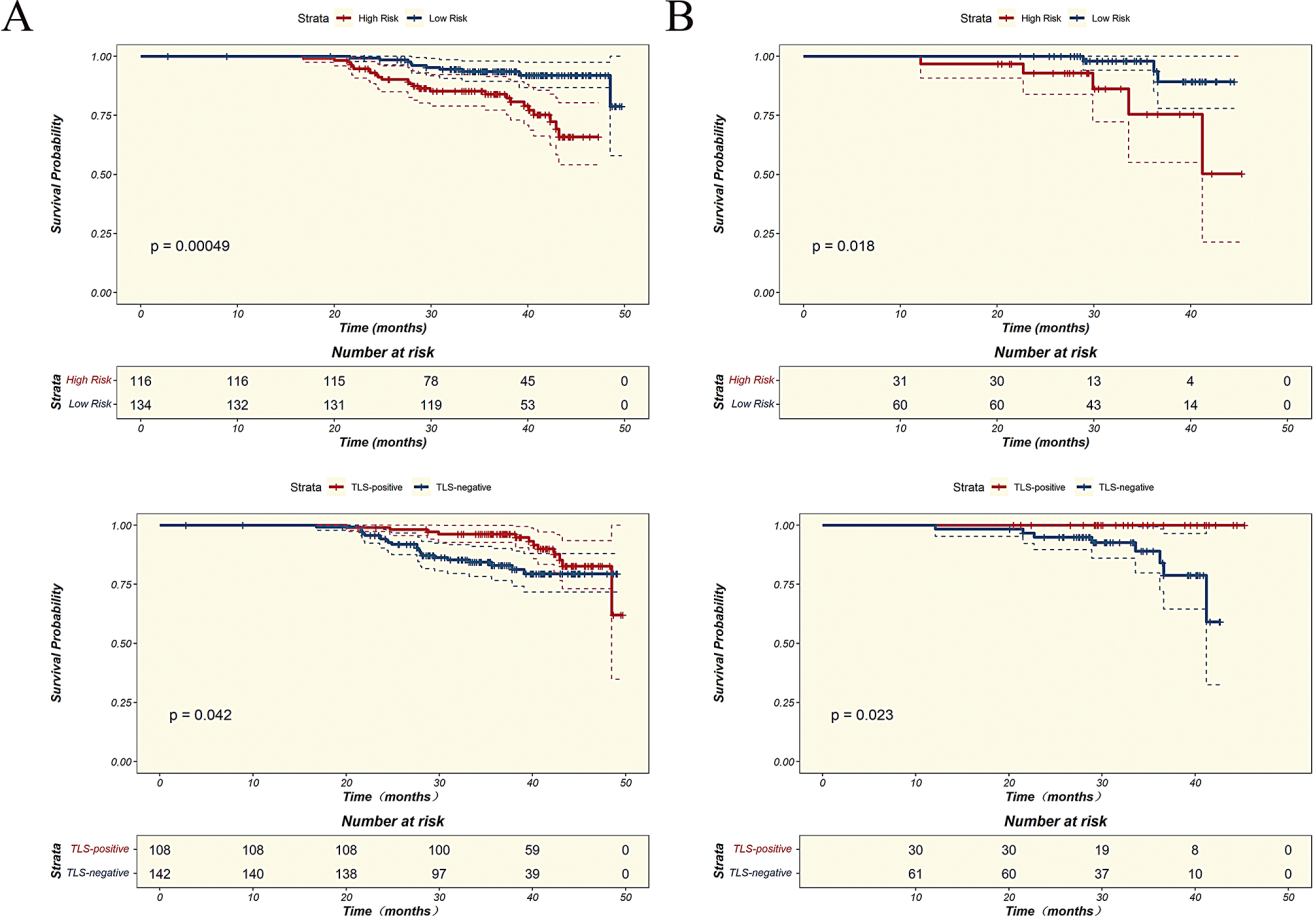
Kaplan-Meier (KM) survival curves for TLS-Positive and TLS-Negative patients, and RAITS high and low risk groups. **(A)** KM survival curves in the training cohort. **(B)** KM survival curves in the validation cohort

Kaplan-Meier (KM) curves for relapse-free survival (RFS) were plotted for TLS-positive and TLS-negative patients in both the training and validation sets. The analysis revealed that TLS-positive patients had significantly higher RFS compared to TLS-negative patients in both the training set (*P* = 0.04) and the validation set (*P* = 0.02) (Fig. 6). The optimal cutoff value of 0.806 for the RAITS model, determined in the training set, was used to classify patients in both the training and validation sets into high-risk and low-risk groups. KM curves for RFS were then plotted for these two groups. The results indicate that RAITS effectively stratifies risk in both the training set (*P*<0.01) and the validation set (*P* = 0.02), demonstrating strong discriminative ability for risk classification. Additionally, significant differences in survival curves between the TLS-positive and TLS-negative groups suggest that RAITS, as a TLS prediction model, is consistent with the actual predictive performance of TLS.

## Discussion

In this study, we developed a multimodal predictive model, RAITS, for identifying TLSs in stage I LUAD based on preoperative CT radiomic features. The model demonstrated robust AUC, sensitivity, and specificity in both the training and external validation cohorts, outperforming traditional unimodal models. In addition to showing strong capabilities in preoperative TLS identification (AUC of 0.78; 95% CI, 0.69–0.88 in the validation cohort), RAITS also exhibits excellent early recurrence risk stratification ability (*P*<0.01 in the training cohort, *P* = 0.02 in the validation cohort).Our findings suggest that RAITS can provide crucial information for clinicians, enabling the development of personalized treatment plans and potentially enhancing treatment outcomes through non-invasive imaging techniques. This model represents a significant advancement in the application of radiomics and deep learning in oncology, offering a valuable tool for improving patient management and prognosis in early-stage LUAD.

We reviewed the current research on TLS and lung adenocarcinoma (LUAD), particularly studies based on real-world data. Remark et al. [15] found that in non-small cell lung cancer (NSCLC), TLS-positive patients had longer survival and lower recurrence rates. Rakaee et al. [16] found that regardless of the (semi)quantitative strategy used, the number of TLS in stage III patients was significantly lower than in stage II patients, and that TLS scoring may improve the TNM staging system for NSCLC. In another study, Gu-Trantien et al. [17] conducted a study in breast cancer patients, which showed that the presence of TLS was associated with better disease-free survival (DFS). Although this study focused on breast cancer, it supports the idea that TLS plays an active role in the tumor immune microenvironment. In addition, a study by NG et al. [18] suggested that TLS was not only a prognostic indicator but could also serve as a predictive biomarker for treatment response. The maturity and abundance of TLS were closely related to the distribution of CD8 + T cells and macrophages, and changes in these immune cells were consistent with changes in TLS characteristics, further supporting the potential of TLS as a predictive biomarker for treatment response. In our study on LUAD, we found that combining radiomic features with deep learning features enabled the early identification of TLS-positive patients before surgery. This provides valuable information for prognostic assessment and supports the development of personalized treatment strategies. Specifically, TLS-positive patients may require earlier genetic testing and PD-1/PD-L1 testing to initiate immunotherapy at an earlier stage, which is significant for improving patient survival and quality of life. In breast cancer research, Li et al. [19] combined radiomics with clinical data to analyze the predictive role of TLS. By constructing a radiomics-clinical integrated model, the study found that the model combining radiomics outperformed the single clinical model in predicting the presence of TLS, demonstrating higher accuracy and better validation performance (training cohort AUC of 0.820, validation cohort AUC of 0.749). Although these studies focus on different types of cancer, they collectively demonstrate the significant potential of radiomics for non-invasive prediction of TLS. Similarly, in intrahepatic cholangiocarcinoma (ICC) research [20], researchers established a radiomics model for predicting intra-tumoral TLS by extracting and selecting 107 radiomic features. The model achieved an AUC of 0.85 in the training cohort, outperforming the independent clinical model (AUC of 0.75) and the single radiomics model (AUC of 0.82). The study further validated the advantage of combining radiomics and clinical features for TLS prediction, achieving strong performance in the external validation cohort (AUC of 0.88). Although these studies have not fully addressed lung adenocarcinoma, they provide important support for the findings in our study. We believe that radiomic features based on tumors and their surrounding tissues will become an important tool for predicting TLS in lung adenocarcinoma in the future, further advancing tumor immunology research and providing strong support for clinical decision-making.

In radiomics studies of LUAD, while the application of imaging features from the tumor periphery is relatively limited, some studies have demonstrated its significance. Recently, Wu et al. [9] confirmed the value of peritumoral CT radiomic features in predicting the prognosis of NSCLC, noting that the peritumoral region is most defined as extending 15 mm, 20 mm, or 30 mm from the tumor boundary. Yang et al. [21] constructed a model by extracting radiomic features from both the tumor interior and a 5 mm peritumoral expansion zone. They reported that the fusion model, based on features from both the tumor interior and the 5 mm peritumoral expansion, achieved AUCs of 0.906 and 0.886 in internal and external validation cohorts, respectively. Similarly, Huang et al. [22] developed a radiomics model based on preoperative CT features from both the tumor and peritumoral regions to predict the primary pathological response in NSCLC patients undergoing neoadjuvant immunotherapy. The results indicated that the comprehensive model incorporating peritumoral features significantly outperformed the unimodal model using only tumor features in predicting pathological response. In our previous study [23], we found that in predicting early recurrence of LUAD, models based on peritumoral radiomic features with 3 mm, 6 mm, and 12 mm expansions did not show improvement with larger peritumoral regions. Therefore, in this study, we focused on a more constrained peritumoral range. Building on these studies, we selected 2 mm, 4 mm, 6 mm, and 8 mm as the peritumoral regions. However, the DeLong test results indicate no significant differences in accuracy between the models constructed with radiomics features from these four regions. Still, there is a significant difference between the RAITS and the Intraperi6mm model (*P* < 0.05) (Supplementary Table 10). We hypothesize that RAITS, by combining features from multiple peritumoral regions, incorporates a broader range of imaging information. Features from different peritumoral regions may capture various aspects of the tumor microenvironment that might not be fully utilized in a single peritumoral region model. This integration of information may enhance the performance of the combined model, making it significantly superior to the single 6 mm region model.

This study used the Radiomics Quality Score (RQS) system or quality control and assessment of our research [24]. According to Wu et al. [9], who conducted a comprehensive evaluation of the value of tumor-peripheral CT radiomics features in predicting the prognosis of LUAD, the median RQS for currently published LUAD tumor-peripheral CT radiomics models is 13 (range: 4–19). The RQS score for RAITS is + 17 (Supplementary Fig. 6). The main limitation is the lack of prospective validation. This indicates that, compared to currently published LUAD tumor-peripheral CT radiomics studies, our research demonstrates higher quality.

However, our study still has some limitations. First, as a retrospective study, selection bias is unavoidable, and the follow-up period is relatively long. Secondly, the performance of RAITS in the validation set reflects that our sample size may need to be further expanded, and the demographic and ethnic diversity of the samples is limited. Future research should be conducted in a larger cohort of LUAD patients. In the future, we will focus on further refining and validating the RAITS, particularly through its application in high-quality, multicenter, prospective studies. Another limitation is that we did not incorporate clinical data for a comprehensive analysis. Although the radiomics model has demonstrated significant potential in predicting tumor microenvironment features, future studies should integrate clinical data, molecular biomarkers, and treatment responses to develop more comprehensive and precise prognostic prediction models. In terms of feature selection for imaging, we found that the characteristics of different peritumoral regions had a significant impact on model performance, especially in the RAITS fusion model, which effectively combines imaging information from multiple peritumoral regions. This suggests that the model not only reflects the tumor’s intrinsic characteristics but may also capture potential biological factors in the tumor microenvironment. The peritumoral region is closely related to biological processes such as tumor cell infiltration, immune responses, and angiogenesis. Therefore, peritumoral features may partially reflect the heterogeneity of the tumor microenvironment. Future research should further explore the relationship between peritumoral features and the tumor microenvironment to provide more biological evidence for personalized treatment and prognostic assessment.

In conclusion, the multimodal predictive model RAITS, based on preoperative CT radiomic features, exhibits strong performance for predicting TLSs in stage I LUAD, surpassing traditional unimodal models. RAITS has the potential to serve as a mature tool for the non-invasive assessment of stage I LUAD, guiding treatment strategies, and facilitating adjuvant therapy planning.

## Electronic supplementary material

Below is the link to the electronic supplementary material.

Supplementary Material 2

Supplementary Material 3

Supplementary Material 4

Supplementary Material 5

Supplementary Material 6

Supplementary Material 7

Supplementary Material 1

Supplementary Material 8

## Data Availability

No datasets were generated or analysed during the current study.

## Abbreviations

LUAD: Lung adenocarcinoma
TLSs: Tertiary lymphoid structures
NSCLC: Non-small cell lung cancer
MPR: Major pathological response
pCR: Pathological complete response
DFS: Disease-free survival
AI: Artificial intelligence
RAITS: Radiomics integrated TLSs system
SD: Standard deviation
Intra: Intra-tumor area
Peri: Peri-tumor area
AUC: Area under the receiver operating characteristics curve
CI: Confidence interval
PPV: Positive predictive values
NPV: Negative predictive values
INTRA: Intra-tumoral signature
IntraPeri2mm: Peritumoral radiomic signature extracted from the 2 mm peritumoral area
IntraPeri4mm: Peritumoral radiomic signature extracted from the 4 mm peritumoral area
IntraPeri6mm: Peritumoral radiomic signature extracted from the 6 mm peritumoral area
IntraPeri8mm: Peritumoral radiomic signature extracted from the 8 mm peritumoral area
DeepL: Radiomics signature constructed by deep learning features
