# Supplementary figures and images for: Preoperative assessment of tertiary lymphoid structures in stage I lung adenocarcinoma using CT radiomics: a multicenter retrospective cohort study
Source: Cancer Imaging. 2024 Dec 18;24:167. doi: 10.1186/s40644-024-00813-5 (PMC11654080; doi:10.1186/s40644-024-00813-5)

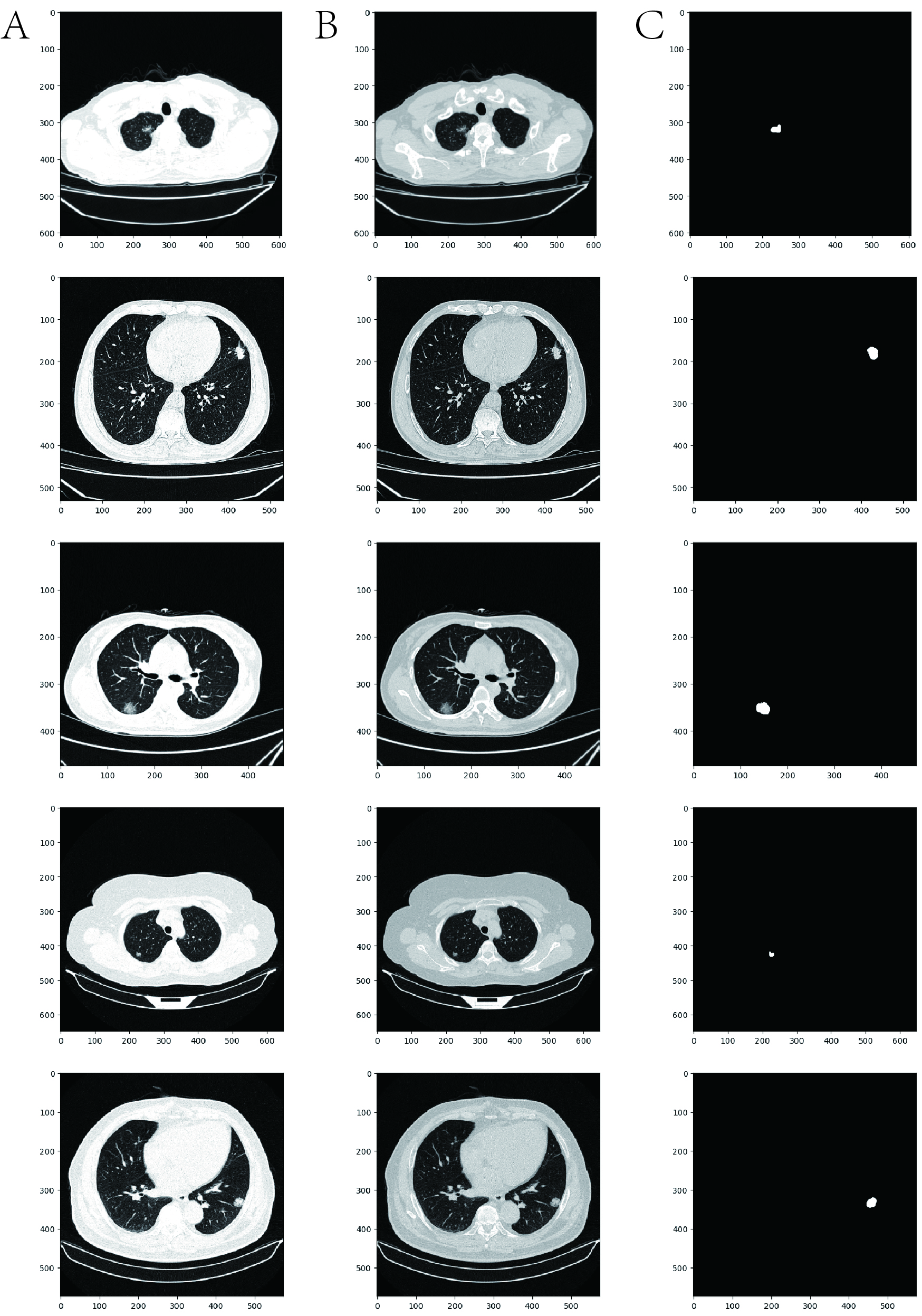

Supplement: Supplementary file 1 — Supplementary Material 2 [file 40644_2024_813_MOESM2_ESM.jpg]

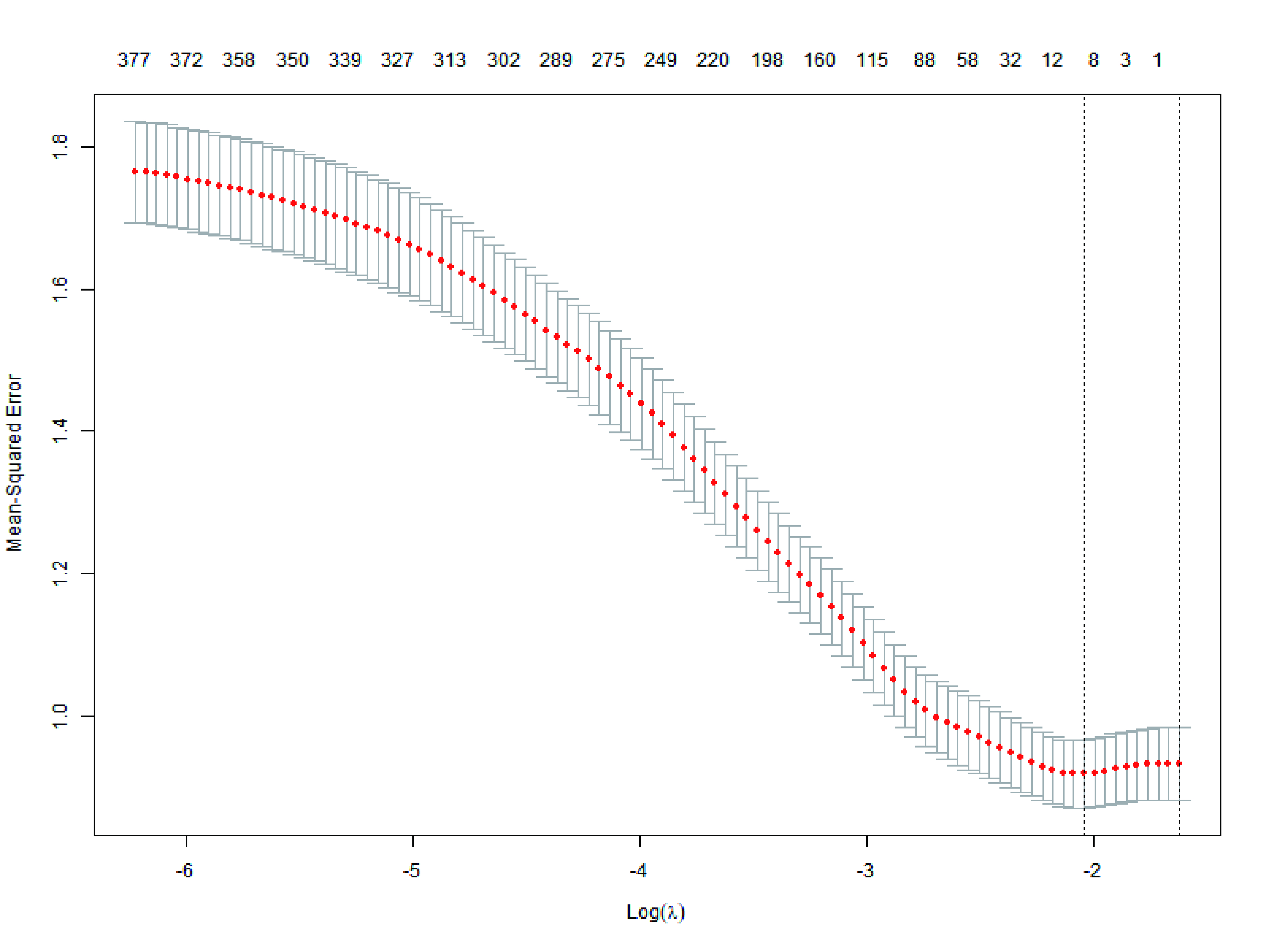

Supplement: Supplementary file 3 — Supplementary Material 4 [file 40644_2024_813_MOESM4_ESM.jpg]

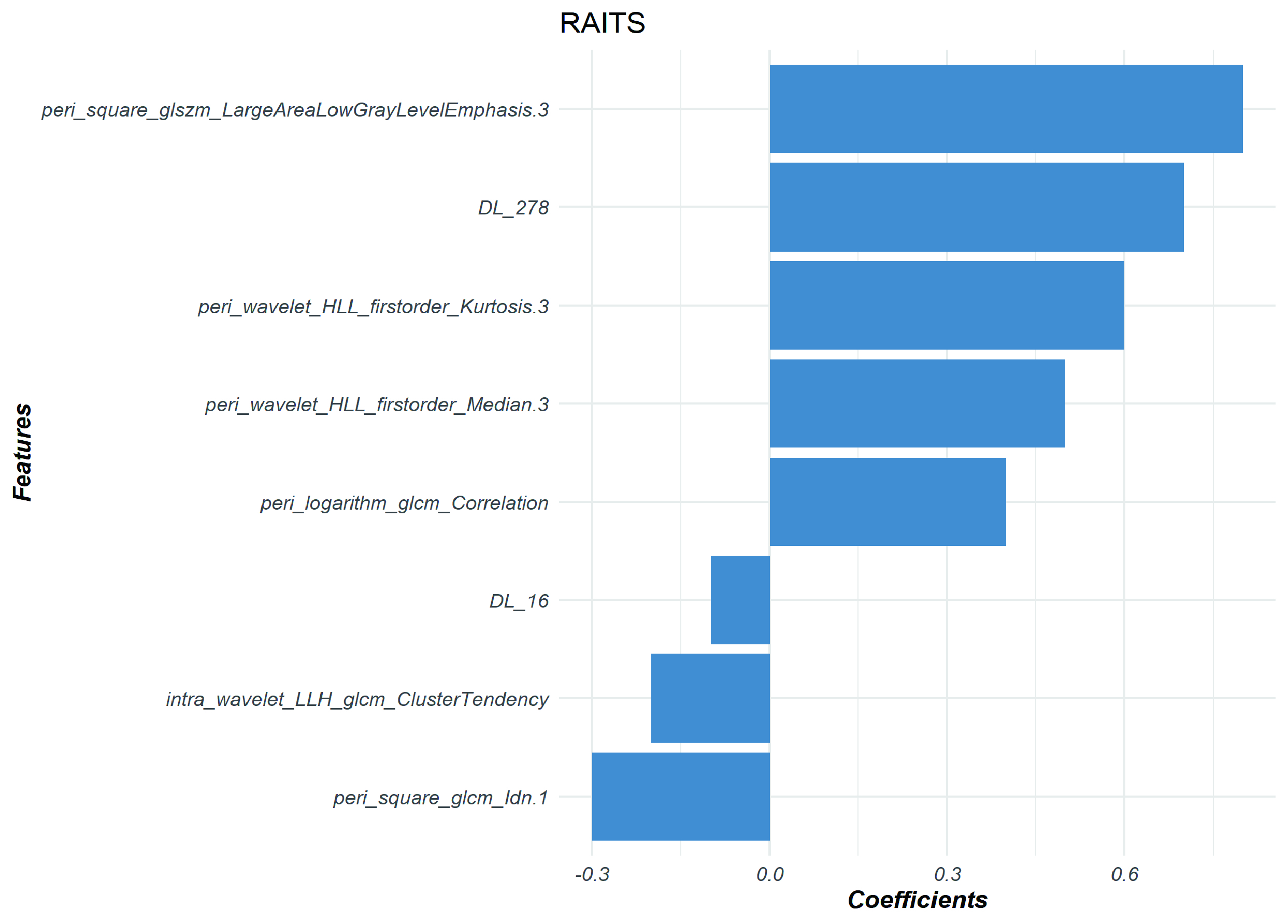

Supplement: Supplementary file 4 — Supplementary Material 5 [file 40644_2024_813_MOESM5_ESM.jpg]

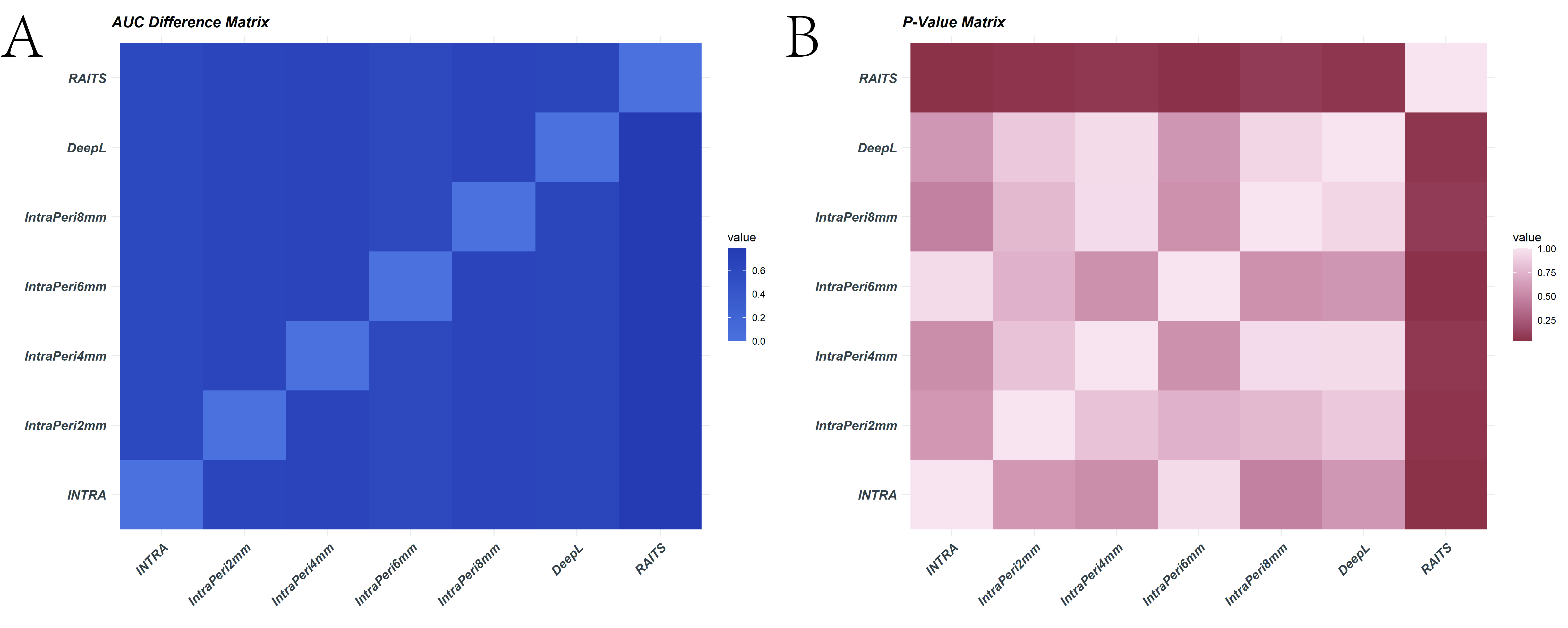

Supplement: Supplementary file 5 — Supplementary Material 6 [file 40644_2024_813_MOESM6_ESM.jpg]

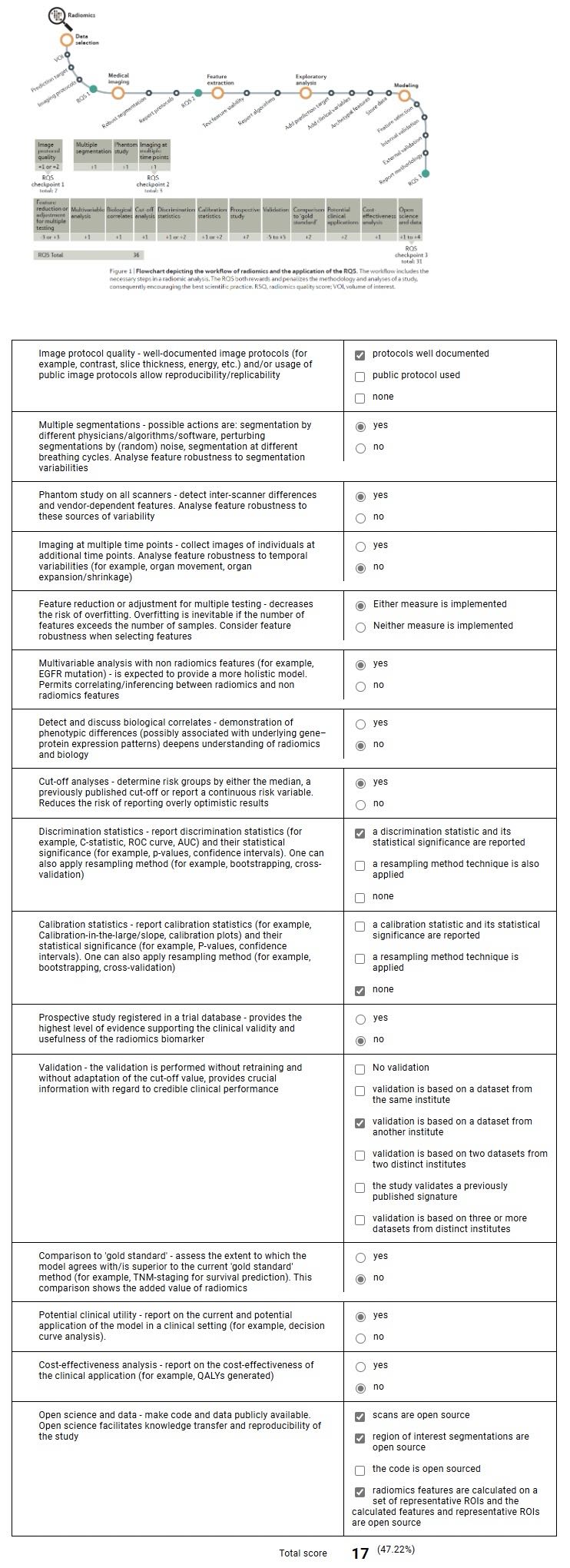

Supplement: Supplementary file 6 — Supplementary Material 7 [file 40644_2024_813_MOESM7_ESM.jpg]
