# Supplementary material for: Preoperative assessment of tertiary lymphoid structures in stage I lung adenocarcinoma using CT radiomics: a multicenter retrospective cohort study
Source: Cancer Imaging. 2024 Dec 18;24:167. doi: 10.1186/s40644-024-00813-5 (PMC11654080; doi:10.1186/s40644-024-00813-5)
Supplement: Supplementary file 7 — Supplementary Material 1 [file 40644_2024_813_MOESM1_ESM.docx]

**Supplementary figure legend**

**Supplementary figure 1** A. Preoperative chest CT images downloaded from the Picture Archiving and Communication System (PACS).B. CT images with voxel dimensions standardized to 0.7 mm × 0.7 mm × 1.5 mm, and window width and level standardized to 1350 HU and -350 HU, respectively. C. Tumor segmentation based on a three-dimensional U-shaped convolutional neural network (3D U-Net).

**Supplementary figure 2** The final features of the six-feature model selected through LASSO-COX regression.

**Supplementary figure 3** LASSO regression path diagram for RAITS.

**Supplementary figure 4** The final feature weights of RAITS selected through LASSO-COX regression.

**Supplementary figure 5** A. Difference matrix of AUC between models. B. *P*-value matrix for differences between models.

**Supplementary figure 6** The RQS score for RAITS.
